# Identifiability of parameters in mathematical models of SARS-CoV-2 infections in humans

**DOI:** 10.1038/s41598-022-18683-x

**Published:** 2022-08-27

**Authors:** Stanca M. Ciupe, Necibe Tuncer

**Affiliations:** 1 Department of Mathematics, Virginia Polytechnic Institute and State University, 225 Stanger Street, Blacksburg, VA 24060 USA; 2 Department of Mathematics, Florida Atlantic University, 777 Glades Road, Boca Raton, FL 33431 USA

**Keywords:** Viral infection, Computational models

## Abstract

Determining accurate estimates for the characteristics of the severe acute respiratory syndrome coronavirus 2 in the upper and lower respiratory tracts, by fitting mathematical models to data, is made difficult by the lack of measurements early in the infection. To determine the sensitivity of the parameter estimates to the noise in the data, we developed a novel two-patch within-host mathematical model that considered the infection of both respiratory tracts and assumed that the viral load in the lower respiratory tract decays in a density dependent manner and investigated its ability to match population level data. We proposed several approaches that can improve practical identifiability of parameters, including an optimal experimental approach, and found that availability of viral data early in the infection is of essence for improving the accuracy of the estimates. Our findings can be useful for designing interventions.

## Introduction

Understanding the upper respiratory tract (URT) kinetics of the severe acute respiratory syndrome coronavirus 2 (SARS-CoV-2) is important for designing public health interventions such as testing, isolation, quarantine, and drug therapies^1–12^. Similarly, understanding the kinetics of SARS-CoV-2 in the lower respiratory tract (LRT) is important for predicting the potential for severe disease, respiratory failure, and/or death^3,13^. Insights into the mechanism of SARS-CoV-2-host interactions and their role in transmission and disease have been found using mathematical models applied to longitudinal data^3–11,14–17^. While these studies are instrumental in determining important parameters (such as SARS-CoV-2 daily shedding and clearance rates, basic reproduction number, the role of innate immune responses in controlling and/or exacerbating the disease), their predictions are limited by the lack of data early in the infection. As such, with few (if any) samples available before viral titers peak, the early virus kinetics and the mechanisms for these early kinetics are uncertain. In this study, we investigate the sensitivity of the predicted outcomes of a within-host model of SARS-CoV-2 infection to the availability of data during different stages of the infection and use our findings to make recommendation.

A German study by Wolfel et al. collected data from nine patients infected early in the pandemic through contact with the same index case^18^. The study showed independent virus replication in upper and lower respiratory tracts^18,19^ suggesting the possibility that virus kinetics, disease stages, and host involvement in control and pathogenesis are dependent on which area of the respiratory tract is homing SARS-CoV-2 at different stages of the disease^20–22^. One shortcoming when evaluating the data in this study comes from the fact that viral RNA was collected only after the patients became symptomatic, with an estimated first data point available on average 5–7 days after infection. Several within-host mathematical models developed and applied to the data set in the Wolfel et al. study have evaluated SARS-CoV-2 parameters, determined the role of innate immune responses, found connections between total RNA and infectious titers, and identified the efficacy of drug therapies^3,6,7^. We are interested in determining how the lack of data early in the infection affects these estimates.

We first developed our own within-host model that does not consider innate immunity explicitly. The model is an expansion of the within-host mathematical models developed for influenza and other respiratory infections^23–26^ that includes both URT and LRT patches. We used the data from Wolfel et al. to estimate pertinent parameters and investigated the sensitivity of the estimated parameters to the presence of data at different stages of infection. To accomplish this, we created virtual data sets that span various stages of the infection and determined how our initial predictions are being influenced by the additional data. Such results may influence our understanding of both viral expansion and the effect of inoculum dose on disease progression.

## Methods

### Mathematical model

**Figure 1:**
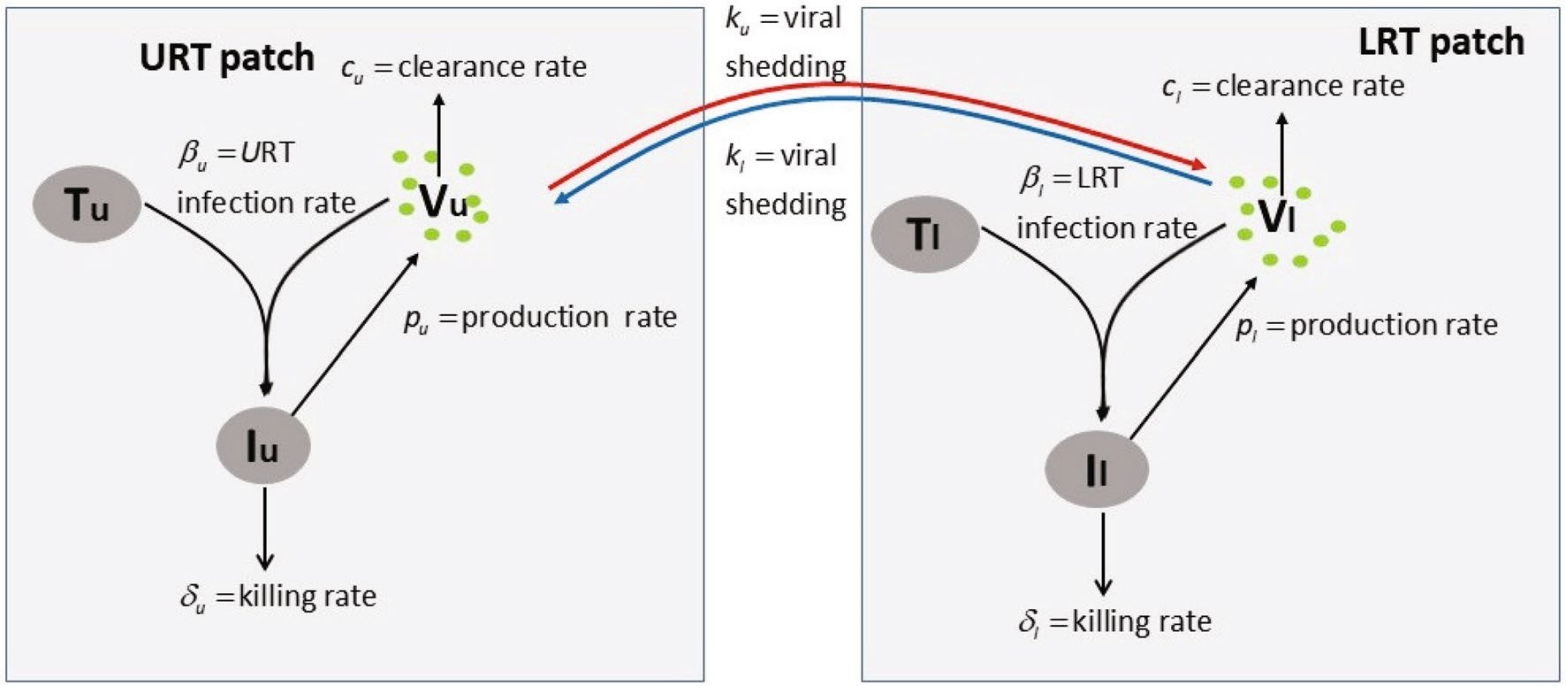
Model diagram.

SARS-CoV-2 virus infects and replicates in epithelial cells of the upper and lower respiratory tract^18^. We model this by developing a two patch within-host model, where the patches are the two respiratory tracts which are linked through virus migration between patches, or viral shedding. Both respiratory tract patches assume interactions between uninfected epithelial cells, $T_j$; infected epithelial cells, $I_j$; and virus homing in tract *j*, $V_j$ at time *t*. Here, $j=\{u,l\}$, with *u* describing the URT patch and *l* describing the LRT patch. Target cells in each patch get infected at rates $\beta _j$ and infected cells produce new virions at rates $p_j$. Infected cells die at rates $\delta _j$ and virus particles are cleared at a linear rate $c_u$ in the upper respiratory tract and in a density dependent manner $c_l V_l/(V_l + K)$ in the lower respiratory tract, where *K* is the viral load in the LRT where the clearance is half maximal. The two patches are linked via the virus populations, with a proportion $k_u$ of $V_u$ migrating from URT to LRT and $k_l$ of $V_l$ migrating from LRT to URT. The model describing these interactions (see Fig. 1) is given by

1 $$\begin{aligned} \begin{aligned} \frac{dT_u}{dt} =&-\beta _u T_uV_u,\\ \frac{dI_u}{dt} =\,&\beta _u T_uV_u,- \delta _u I_u, \\ \frac{dV_u}{dt} =\,&p_u I_u - c_uV_u + k_l V_l, \\ \frac{dT_l}{dt} =\,&-\beta _l T_l V_l,\\ \frac{dI_l}{dt} =\,&\beta _lT_l V_l- \delta _l I_l, \\ \frac{dV_l}{dt} =\,&p_l I_l - c_l \frac{V_l}{V_l+K}V_l + k_u V_u.\\ \end{aligned} \end{aligned}$$

We model the initial conditions of the system Eq. (1) as follows. We assume that all epithelial cells in the URT and LRT patches are susceptible to virus infection. When infection occurs, it results in a small initial virus inoculum which homes in the URT alone. Under these assumptions, system Eq. (1) is subject to initial conditions

2 $$\begin{aligned} \begin{aligned} T_u(0)&= T_u^0, \ I_u(0) = 0, \ V_u(0) = V_0, \\ T_l(0)&= T_l^0, \ I_l(0) = 0, \ V_l(0) = 0, \end{aligned} \end{aligned}$$

where $V_0$ is the viral inoculum. We aim to determine the dynamics of system Eq. (1) over time for model parameters that explain URT and LRT tract data in a single patient (patient A) and in the population data (all nine patients) from^18^.

### Parameter estimation

#### Patient data

**Figure 2:**
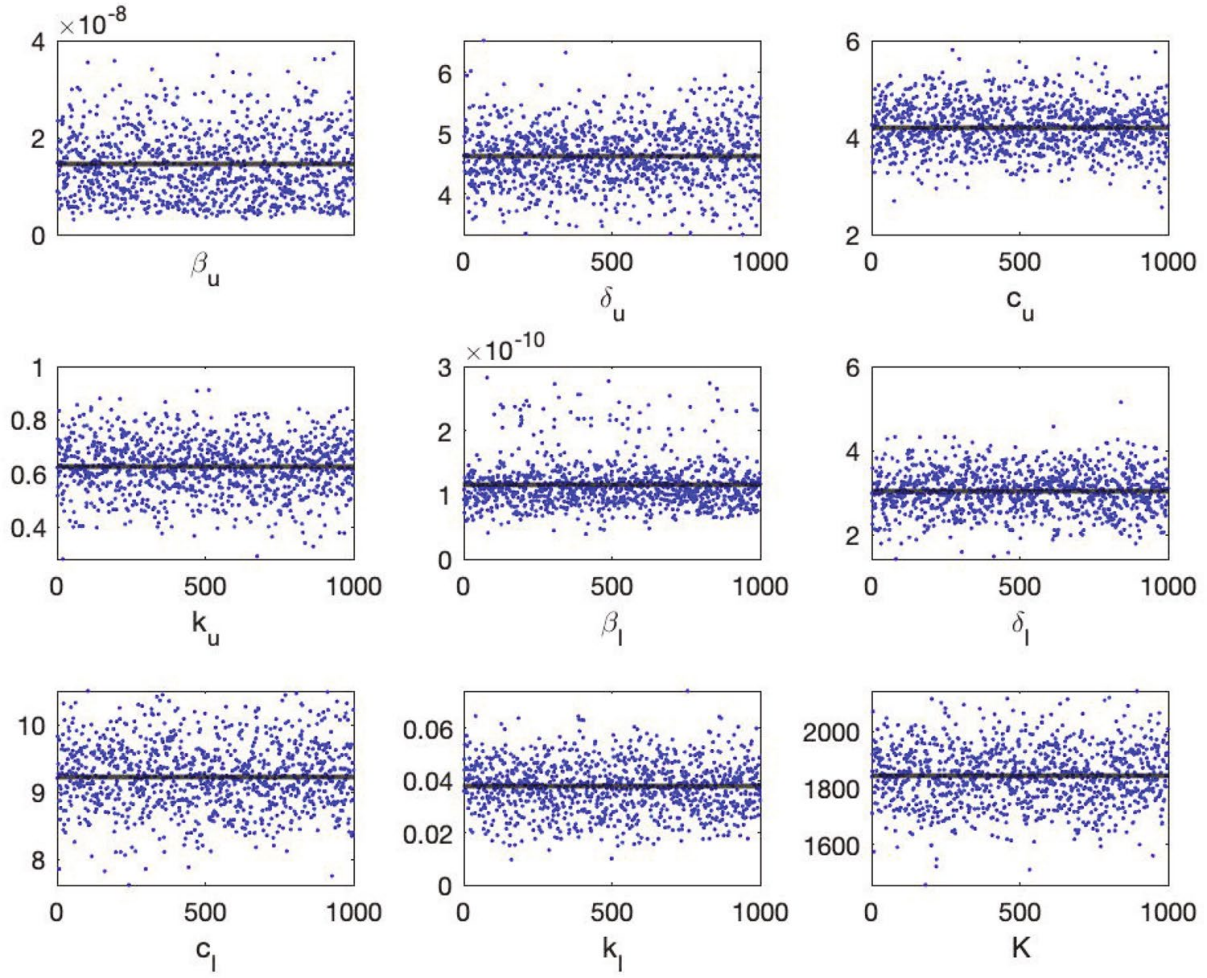
The Markov chain of the within-host model Eq. (7)’s parameters obtained when the model is fitted to the population data. Every 1000th point of $10^6$ iterations are shown. The black line shows the mean of the chain.

In January 2020, nine patients tested positive for COVID-19 in a single-source outbreak in Bavaria, Germany^19^. Early detection allowed for rapid contact tracing, testing, and monitoring of the affected community: young healthy professionals in their mid-thirties. A followup study published time series for the post symptoms virus data isolated from oral-and nasopharyngeal throat swabs (in copies per swabs) and from sputum samples (in RNA copies per mL) for the same patient population over their entire course of disease. The patients’ throat swabs and sputum data (Fig. 2 of ^18^) were obtained through personal communication with the authors. Since we know the incubation period for each patient^19^ (see Table 1), we assume time zero in our study to be the day of infection for the patients in Ref.^18^.

**Table 1:** Incubation periods estimated in Ref.^19^.

Patient id	A	B	C	D	E	F	G	H	I
Incubation period (days)	2.5	4	1	4	4	4	2	4.5	7

#### Identifiability analysis

Using the URT and LRT viral load data, we aim to determine the unknown parameters $p = \{\beta _u,\delta _u,p_u,c_u,k_l,\beta _l, \delta _l,p_l,c_l,K,k_u\}$ of the within-host model Eq. (1). Before attempting to estimate the within-host model parameters using noisy laboratory data, it is crucial to analyze whether the model is structurally identifiable. Specifically, we need to know if the within-host model Eq. (1) is structured to reveal its parameters from upper and lower viral load observations. We approach this problem in an ideal setting where we assume that the observations are known for every $t>0$ and they are not contaminated with any noise. This analysis is called structural identifiability^27^.

The observed data in Wolfel et al.^18^ is modeled in the within-host model Eq. (1) by variables $V_u$ and $V_l$, which account for the upper and lower respiratory tract viral titers. We denote these observed variable as

$$\begin{aligned} y_1(t) = V_u(t) \quad \text {and} \quad y_2(t) = V_l(t). \end{aligned}$$

First, we give the definition of structural identifiability in terms of the observed variables $y_1(t)$ and $y_2(t)$^27–31^.

##### Definition 1

Let $p$ and $q$ be the two distinct vectors of within-host model Eq. (1) parameters. We say that the within-host model is structurally (globally) identifiable if and only if

$$\begin{aligned} y_1(t,p) = y_1(t,q) \quad \text {and} \quad y_2(t,p) = y_2(t,q)\quad \implies \quad p= q . \end{aligned}$$

Simply put, we say that the within-host model Eq. (1) is structurally identifiable if two identical observation are only possible for identical parameters. We perform the structural identifiability analysis via differential algebra approach. The first step in this approach is eliminating the unobserved state variables from the within-host model Eq. (1). The reason for eliminating the unobserved state variables is to obtain a system which only involves the observed states and model parameters. Since this is a complex procedure, we use DAISY^32^ and obtain the following system

3 $$\begin{aligned} \begin{aligned}{}&\displaystyle \frac{d^3 y_1}{dt^3}y_1 - \displaystyle \frac{d^2 y_1}{dt^2}\displaystyle \frac{d y_1}{dt} + \displaystyle \frac{d^2 y_1}{dt^2} y_1^2 \beta _u + \displaystyle \frac{d^2 y_1}{dt^2} y_1(c_u + \delta _u) - \left( \displaystyle \frac{d y_1}{dt}\right) ^2 (c_u + \delta _u) + \displaystyle \frac{d y_1}{dt} \displaystyle \frac{d y_2}{dt}k_l \\& \quad + \displaystyle \frac{d y_1}{dt} y_1^2 \beta _u (c_u + \delta _u) +\displaystyle \frac{d y_1}{dt} y_2 \delta _u k_l - \displaystyle \frac{d^2y_2}{dt^2}y_1k_l - \displaystyle \frac{d y_2}{dt} y_1^2 \beta _u k_l - \displaystyle \frac{d y_2}{dt}y_1 \delta _u k_l + y_1^3 \beta _u c_u \delta _u - y_1^2 y_2 \beta _u \delta _u k_l = 0.&\end{aligned} \end{aligned}$$

and

4 $$\begin{aligned} \begin{aligned}{}&-\displaystyle \frac{d^2 y_1}{dt^2} y_2^5 k_u - 4 \displaystyle \frac{d^2 y_1}{dt^2} y_2^4 K k_u - 6 \displaystyle \frac{d^2 y_1}{dt^2} y_2^3 K^2 k_u - 4 \displaystyle \frac{d^2 y_1}{dt^2} y_2^2 K ^3 k_u -\displaystyle \frac{d^2 y_1}{dt^2} y_2 K^4 k_u + \displaystyle \frac{d y_1}{dt} \displaystyle \frac{d y_2}{dt} y_2^4 k_u \\&\quad + 4 \displaystyle \frac{d y_1}{dt} \displaystyle \frac{d y_2}{dt} y_2^3 K k_u + 6 \displaystyle \frac{d y_1}{dt} \displaystyle \frac{d y_2}{dt} y_2^2 K^2 k_u + 4 \displaystyle \frac{d y_1}{dt} \displaystyle \frac{d y_2}{dt} y_2 K^3 k_u + \displaystyle \frac{d y_1}{dt} \displaystyle \frac{d y_2}{dt} K^4 k_u \\&\quad - \displaystyle \frac{d y_1}{dt} y_2^6 \beta _l k_u + \displaystyle \frac{d y_1}{dt} y_2^5 k_u ( - 4 \beta _l K - \delta _l) + 2 \displaystyle \frac{d y_1}{dt} y_2^4 K k_u ( - 3 \beta _l K - 2 \delta _l) + 2 \displaystyle \frac{d y_1}{dt} y_2^3 K^2 k_u ( - 2 \beta _l K - 3 \delta _l) \\&\quad + \displaystyle \frac{d y_1}{dt} y_2^2 K^3 k_u ( - \beta _l K - 4 \delta _l) - \displaystyle \frac{d y_1}{dt} y_2 \delta _l K^4 k_u + \displaystyle \frac{d^3 y_2}{dt^3} y_2^5 + 4 \displaystyle \frac{d^3 y_2}{dt^3} y_2^4 K + 6 \displaystyle \frac{d^3 y_2}{dt^3} y_2^3 K^2 + 4 \displaystyle \frac{d^3 y_2}{dt^3} y_2^2 K^3 \\&\quad + \displaystyle \frac{d^3 y_2}{dt^3} y_2 K^4 - \displaystyle \frac{d^2 y_2}{dt^2} \displaystyle \frac{d y_2}{dt} y_2^4 - 4 \displaystyle \frac{d^2 y_2}{dt^2} \displaystyle \frac{d y_2}{dt} y_2^3 K - 6 \displaystyle \frac{d^2 y_2}{dt^2} \displaystyle \frac{d y_2}{dt} y_2^2 K^2 - 4 \displaystyle \frac{d^2 y_2}{dt^2} \displaystyle \frac{d y_2}{dt} y_2 K^3 - \displaystyle \frac{d^2 y_2}{dt^2} \displaystyle \frac{d y_2}{dt} K^4 \\&\quad + \displaystyle \frac{d^2 y_2}{dt^2} y_2^6 \beta _l + \displaystyle \frac{d^2 y_2}{dt^2} y_2^5 (4 \beta _l K + \delta _l) + 2 \displaystyle \frac{d^2 y_2}{dt^2} y_2^4 K (3 \beta _l K + 2 \delta _l) + \displaystyle \frac{d^2 y_2}{dt^2} y_2^3 K (4 \beta _l K^2 + c_l + 6 \delta _l K) \\&\quad + \displaystyle \frac{d^2 y_2}{dt^2} y_2^2 K^2 (\beta _l K^2 + 2 c_l + 4 \delta _l K) + \displaystyle \frac{d^2 y_2}{dt^2} y_2 K^3 (c_l + \delta _l K) - \displaystyle \frac{d y_2}{dt}^2 y_2^4 \delta _l - 4 \displaystyle \frac{d y_2}{dt}^2 y_2^3 \delta _l K \\&\quad + 3 \displaystyle \frac{d y_2}{dt}^2 y_2^2 K ( - c_l - 2 \delta _l K) - 4 \displaystyle \frac{d y_2}{dt}^2 y_2 K^2 (c_l + \delta _l K) - \displaystyle \frac{d y_2}{dt}^2 K^3 (c_l + \delta _l K) + \displaystyle \frac{d y_2}{dt} y_1 y_2^4 \delta _l k_u \\&\quad + 4 \displaystyle \frac{d y_2}{dt} y_1 y_2^3 \delta _l K k_u + 6 \displaystyle \frac{d y_2}{dt} y_1 y_2^2 \delta _l K^2 k_u + 4 \displaystyle \frac{d y_2}{dt} y_1 y_2 \delta _l K^3 k_u + \displaystyle \frac{d y_2}{dt} y_1 \delta _l K^4 k_u \\&\quad + \displaystyle \frac{d y_2}{dt} y_2^6 \beta _l \delta _l + 4 \displaystyle \frac{d y_2}{dt} y_2^5 \beta _l \delta _l K + \displaystyle \frac{d y_2}{dt} y_2^4 (\beta _l c_l K + 6 \beta _l \delta _l K^2 - c_l \delta _l) + 2 \displaystyle \frac{d y_2}{dt} y_2^3 K (\beta _l c_l K + 2 \beta _l \delta _l K^2 - c_l \delta _l) \\&\quad + \displaystyle \frac{d y_2}{dt} y_2^2 K^2 (\beta _l c_l K + \beta _l \delta _l K^2 - c_l \delta _l) - y_1 y_2^6 \beta _l \delta _l k_u - 4 y_1 y_2^5 \beta _l \delta _l K k_u - 6 y_1 y_2^4 \beta _l \delta _l K^2 k_u \\&\quad - 4 y_1 y_2^3 \beta _l \delta _l K^3 k_u - y_1 y_2^2 \beta _l \delta _l K^4 k_u + y_2^6 \beta _l c_l \delta _l + 3 y_2^5 \beta _l c_l \delta _l K + 3 y_2^4 \beta _l c_l \delta _l K^2 + y_2^3 \beta _l c_l \delta _l K^3 =&0.\\ \end{aligned} \end{aligned}$$

Equations (3) and (4) are called input–output equations of within-host model Eq. (1), which are differential polynomials involving the observed state variables $y_1=V_u(t)$ and $y_2=V_l(t)$ and the within-host model parameters. Note that solving input–output equations Eqs. (3) and (4) is equivalent to solving the within-host model Eq. (1) for the state variables $V_u(t)$ and $V_l(t)$. For identifiability analysis, it is crucial that the input-output equations are monic, i.e. the leading coefficient is 1. It is clear that the input–output equation Eq. (3) is monic, and the input–output equation Eq. (4) can be made monic by dividing the coefficients with the coefficient of the leading term, which is $k_u$. As a result, the definition of the structural identifiability within differential algebra approach which involves input–output equations takes the following form^27–30^.

##### Definition 2

Let $c(p)$ denote the coefficients of the input-output equations, Eqs. (3) and (4), where $p$ is the vector of model parameters. We say that the within-host model Eq. (1) is structured to reveal its parameters from the observations if and only if

$$\begin{aligned} c(p) = c(q) \quad \implies \quad p= q. \end{aligned}$$

Suppose $p = \{\beta _u,\delta _u,p_u,c_u,k_l,\beta _l, \delta _l,p_l,c_l,K,k_u\}$ and $q = \{{\hat{\beta }}_u,{\hat{\delta }}_u,{\hat{p}}_u,{\hat{c}}_u,{\hat{k}}_l,{\hat{\beta }}_l, {\hat{\delta }}_l,{\hat{p}}_l,{\hat{c}}_l,{\hat{K}},{\hat{k}}_u\}$ are two parameter sets of the within-host model which produced the same observations. This can only happen if the coefficients of the input-output Eqs. (3) and (4) are the same. Hence, if $c(p )$ denote the coefficients of the corresponding monic polynomial of input–output equations, we solve $c(p ) = c(q)$ to obtain

5 $$\begin{aligned} \beta _u = {\hat{\beta }}_u,\, \delta _u = {\hat{\delta }}_u,\, c_u = {\hat{c}}_u,\, k_u = {\hat{k}}_u, \,\beta _l = {\hat{\beta }}_l,\, \delta _l = {\hat{\delta }}_l,\, c_l = {\hat{c}}_l,\, K = {\hat{K}},\, k_l = {\hat{k}}_l. \end{aligned}$$

The solution set (5) means that the parameters, $\beta _u, \delta _u, c_u, k_u, \beta _l,\delta _l,c_l,K$ and $k_l$ can be identified uniquely. However, parameters $p_u$ and $p_l$ both disappear from the input–output Eqs. (3) and (4). It is easier to see the reason behind this by scaling the unobserved state variables of the within-host model Eq. (1) with a postive scalar $\sigma >0$. Hence, $(\sigma T_u,\sigma I_u, V_u, \sigma T_l,\sigma I_l,V_l) = ({\hat{T}}_u,{\hat{I}}_u, V_u, {\hat{T}}_l,{\hat{I}}_l,V_l)$ will solve the following system

6 $$\begin{aligned} \begin{aligned} \frac{d{\hat{T}}_u}{dt} =&-\beta _u {\hat{T}}_uV_u,\\ \frac{d{\hat{I}}_u}{dt} =\,&\beta _u {\hat{T}}_uV_u,- \delta _u {\hat{I}}_u, \\ \frac{d V_u}{dt} =\,&{\hat{p}}_u {\hat{I}}_u - c_uV_u + k_l V_l, \\ \frac{d{\hat{T}}_l}{dt} =&-\beta _l {\hat{T}}_l V_l,\\ \frac{d {\hat{I}}_l}{dt} =\,&\beta _l{\hat{T}}_l V_l- \delta _l {\hat{I}}_l, \\ \frac{dV_l}{dt} =\,&{\hat{p}}_l {\hat{I}}_l - c_l \frac{V_l}{V_l+K}V_l + k_u V_u,\\ \end{aligned} \end{aligned}$$

where ${\hat{p}}_u = \frac{p_u}{\sigma }$ and ${\hat{p}}_l = \frac{p_l}{\sigma }.$ Since $\sigma > 0$ was arbitrary and the observations do not give information about the scaling parameter $\sigma$, the parameters $p_u$ and $p_l$ can not be identified from the viral load in the URT and LRT tracts. We conclude that the within-host model Eq. (1) is not identifiable. We summarize the structural identifiability result in the following Proposition 1.

##### Proposition 1

*The within-host model Eq*. (1) *is not structured to reveal its parameters from the observations of viral load in upper and lower respiratory tracts*. *The parameters* $p_u$ *and* $p_l$ *are not identifiable and only the parameters* $\beta _u,\delta _u,c_u,k_u,\beta _l,\delta _l,c_l,K,k_l$ *can be identified*.

To obtain a structurally identifiable model from the $V_u$ and $V_l$ observations, we scale the unobserved state variables with ${\hat{T}}_u = p_u T_u, \,{\hat{I}}_u = p_u I_u, \, {\hat{T}}_l = p_l T_l, \, {\hat{I}}_l = p_l I_l$ and obtain the following scaled within-host model

7 $$\begin{aligned} \begin{aligned} \frac{d{\hat{T}}_u}{dt} =&-\beta _u {\hat{T}}_u V_u,\\ \frac{d{\hat{I}}_u}{dt} =\,&\beta _u {\hat{T}}_u V_u,- \delta _u {\hat{I}}_u, \\ \frac{dV_u}{dt} =\,&{\hat{I}}_u - c_uV_u + k_l V_l, \\ \frac{d{\hat{T}}_l}{dt} =&-\beta _l {\hat{T}}_l V_l,\\ \frac{d{\hat{I}}_l}{dt} =\,&\beta _l {\hat{T}}_l V_l- \delta _l {\hat{I}}_l, \\ \frac{dV_l}{dt} =\,&{\hat{I}}_l - c_l \frac{V_l}{V_l+K}V_l + k_u V_u.\\ \end{aligned} \end{aligned}$$

##### Proposition 2

*The scaled within-host model Eq*. (7) *is structured to reveal its parameters from the observations of viral load in upper and lower respiratory tracts*. *All the parameters*

$$\begin{aligned} \beta _u,\delta _u,c_u,k_u,\beta _l,\delta _l,c_l,K,k_l \end{aligned}$$

*can be identified, hence the within-host model Eq*. (7) *is globally identifiable*.

### Data fitting

#### Parameter values

We assume that the upper respiratory tract susceptible population is $T_0^u=4\times 10^8$ epithelial cells, as in influenza studies^23^. This estimate was obtained by assuming a URT surface in adults of 160 cm$^2$^33^ and an epithelial cell’s surface area of $2 \times 10^{-11} - 4 \times 10^{-11}$ m$^2$^34^. We use a similar method to estimate the target cell population in the LRT. The lung’s surface area of 70 m$^2$ (with range between 35 m$^2$ and 180 m$^2$)^35^ is composed of gas exchange regions (aveoli), and of conducting airways (trachea, bronchi, bronchioles). Since the gas exchange region is affected by SARS-Cov-2 only in severe cases^20^ we ignore it, and restrict the LRT compartment to the conducting airways whose surface area is $2471 \pm 320$ cm$^2$^36^. Therefore, we obtain an initial epithelial cell target population in the LRT of $T_l^0 = 6.25 \times 10^{9}$ epithelial cells. If we assume that viral production rates are $p_u=50$ and $p_l=32$ per day then, after scaling, we have initial target cell populations in the URT and LRT of ${\hat{T}}_{u}^0 = 2 \times 10^{10}$ epithelial cells and ${\hat{T}}_l^0 = 2 \times 10^{11}$ epithelial cells. The other initial conditions are unaffected by scaling and are set at ${\hat{I}}_u^0= {\hat{I}}_L^0=0$, ${\hat{V}}_u^0= 0.1$ and ${\hat{V}}_L^0=0$, where the virus inoculum of ${\hat{V}}_u^0 = 0.1$ cp/ml is set below the reported limit of quantification of $10^2$ cp/ml^18^. Lastly, the incubation periods were estimated in Ref.^19^ and are listed in Table 1.

#### Bayesian parameter estimation

During the data collection process, observations are perturbed with noise. Hence, the URT and LRT viral load deviates from the smooth trajectory of the observations $y_1(t)$ and $y_2(t)$ at measurement times. We represent measurement error using the following statistical model

8 $$\begin{aligned} \begin{aligned}{}&V_u^{data}(t_i) = y_1(t_i, {\hat{p}}) +\epsilon _i\quad i=1,2,\ldots ,n_u;\\&V_l^{data}(t_j) = y_2(t_j, {\hat{p}}) +\epsilon _j\quad j=1,2,\ldots ,n_l; \end{aligned} \end{aligned}$$

where ${{\hat{p}}}$ is the true parameter vector assumed to generate the data, and the random variables $\epsilon _i$ and $\epsilon _j$ are assumed to be Gaussian with mean zero and standard deviation $\sigma$.

We use Bayesian inference and Markov Chain Monte Carlo (MCMC) to determine the remaining nine parameters of the model Eq. (7)

$$\begin{aligned} p=\{\beta _u, \delta _u, c_u, k_u, \beta _l, \delta _l, c_l, K, k_l\}. \end{aligned}$$

Bayesian inference treats model parameters as random variables and seeks to determine the parameters’ posterior distribution, where the term “posterior” refers to data-informed distributions. The posterior densities are determined using Bayes’ theorem, which defines them as the normalised product of the prior density and the likelihood. Let $\pi (p| {\mathcal {D}})$ denote the probability distribution of the parameter $p$ given the data ${\mathcal {D}} = \Big (V_u(t_i), V_l(t_j)\Big )$, then the Bayes theorem states that

$$\begin{aligned} \pi (p| {\mathcal {D}}) = \displaystyle \frac{\pi ({\mathcal {D}} |p) \pi (p)}{\pi ({\mathcal {D}})}, \end{aligned}$$

where $\pi (p)$ is the prior parameter distribution and $\pi ({\mathcal {D}})$ is a constant which is usually considered to be a normalization constant so that the posterior distribution is indeed a probability density function (pdf), i.e. its integral equals to 1. The likelihood function $\pi ({\mathcal {D}} |p)$ gives the probability of observing the measurements ${\mathcal {D}}$ given that the parameter values is $p$. In terms of the within-host model Eq. (7) and the laboratory data Eq. (8), the likelihood function $\pi ({\mathcal {D}} |p)$ takes the following form

9 $$\begin{aligned} \begin{aligned} \pi ({\mathcal {D}} |p) =&\displaystyle \prod _{i=1}^{n_u} \displaystyle \frac{1}{\sqrt{2}\pi \sigma ^2} e^{-\displaystyle \frac{1}{\sigma ^2}\left( \log _{10}(V_u(t_i))-\log _{10}(V_u^{data}(t_i)\right) ^2} \\&\quad \times \displaystyle \prod _{j=1}^{n_l} \displaystyle \frac{1}{\sqrt{2}\pi \sigma ^2} e^{-\displaystyle \frac{1}{\sigma ^2}\left( \log _{10}(V_l(t_j))-\log _{10}(V_l^{data}(t_j)\right) ^2}. \end{aligned} \end{aligned}$$

The ultimate goal is to determine the posterior distributions of the parameters in the light of laboratory data. To approximate the posterior distributions, we use the MCMC method introduced in Refs.^37,38^. MCMC methods generate a sequence of random samples $p_1,\ p_2,\ldots , p_N$ whose distribution asymptotically approaches the posterior distribution for size *N*. The random walk Metropolis algorithm is one of the most extensively used MCMC algorithms. The Metropolis algorithm starts at position $p_i$, then the Markov chain generates a candidate parameter value $p_*$ from the proposal distribution, and the algorithm accepts or rejects the proposed value based on probability $\alpha$ given by

$$\begin{aligned} \alpha = \min \left( \displaystyle 1, \frac{\pi (p_*)}{\pi (p_i)}\right) . \end{aligned}$$

As with the Metropolis algorithm, the essential feature of MCMC approaches is the formulation of a proposal distribution and an accept–reject criteria. In this paper, we employ the Delayed Rejection Adaptive Metropolis, (DRAM^37^) and use the MATLAB toolbox provided by the same authors^39^. In comparison to other Metropolis algorithms, the Markov chain constructed with DRAM is robust and converges rapidly (see Fig. 2).

The two patch within-host model Eq. (7) is novel, hence we do not have any prior information regarding model parameters. We determine the prior distributions by fitting the structurally identifiable within-host model Eq. (7) to patient A’s data and to the population data (all nine patients). The prior distributions $\pi (p)$ are then defined as a normal distribution with a mean equal to the fitted value and variance equal to $\sigma ^2$, $\pi (p) \sim N (\mu , \sigma ).$ Table 2 shows the obtained prior distribution of each parameter for patient A and population data, together with the lower and upper bounds of the prior $\pi (p)$.

**Table 2:** Parameters for the within-host model Eq. (7) are listed together with their lower and upper bounds for the priors. Prior distributions are normally distributed with mean equal to the fitted value and variance, $\sigma ^2$.

Parameter	Description	Prior $\pi (p)$	Patient A	Population
		Min–Max	$\pi (p) \sim {\mathcal {N}} (\mu , \sigma )$	$\pi (p) \sim {\mathcal {N}} (\mu , \sigma )$
$\beta _u$	Viral infectivity in URT	$(10^{-12}, 10^{-7})$	${\mathcal {N}} (1.1 \times 10^{-8},10^{-8})$	${\mathcal {N}} (8.9 \times 10^{-9},10^{-8})$
$\beta _l$	Viral infectivity in LRT	$(10^{-12}, 10^{-7})$	${\mathcal {N}} (3.9 \times 10^{-8},10^{-10})$	${\mathcal {N}} (9.3 \times 10^{-11},10^{-10})$
$\delta _u$	Infected cell decay rate in URT	(0, 50)	${\mathcal {N}} (4.88,0.5)$	${\mathcal {N}} (4.64,0.5)$
$\delta _l$	Infected cell decay rate in LRT	(0, 50)	${\mathcal {N}} (5.59,0.5)$	${\mathcal {N}} (2.99,0.5)$
$c_u$	Viral decay rate in URT	(0, 30)	${\mathcal {N}} (2.88,0.5)$	${\mathcal {N}} (4.27,0.5)$
$c_l$	Viral decay rate in LRT	(0, 30)	${\mathcal {N}} (11.43,0.5)$	${\mathcal {N}} (9.21,0.5)$
*K*	$V_l$ where loss is half-maximal	(0, 3000)	${\mathcal {N}} (910,100)$	${\mathcal {N}} (1840,100)$
$k_u$	Shedding into LRT	$(10^{-6}, 1)$	${\mathcal {N}} (0.24,0.1)$	${\mathcal {N}} (0.62,0.1)$
$k_l$	Shedding into URT	$(10^{-6}, 1)$	${\mathcal {N}} (0.0008,0.0001)$	${\mathcal {N}} (0.036,0.01)$

## Results

### Viral dynamics

To study the kinetics of SARS-CoV-2 in the upper and lower respiratory tracts we developed a two patch within-host model Eq. (1) that assumed viral shedding between the two patches. To ensure structural identifiability, we rescaled our equations by removing the non-identifiable parameters $p_u$ and $p_l$ (see “Identifiability analysis” section in “Materials and methods”). The resulting model Eq. (7) was validated against SARS-CoV-2 RNA data from throat swabs and sputum samples collected from an infectious event with the same index case early in the pandemic^18^. We used Bayesian parameter estimation with the viral samples in URT and LRT from a single individual (patient A) and the entire population (nine individuals) and approximated posterior distributions with $N=10^6$ Markov chain iterations (see “Data fitting” section in “Materials and methods”).

**Figure 3:**
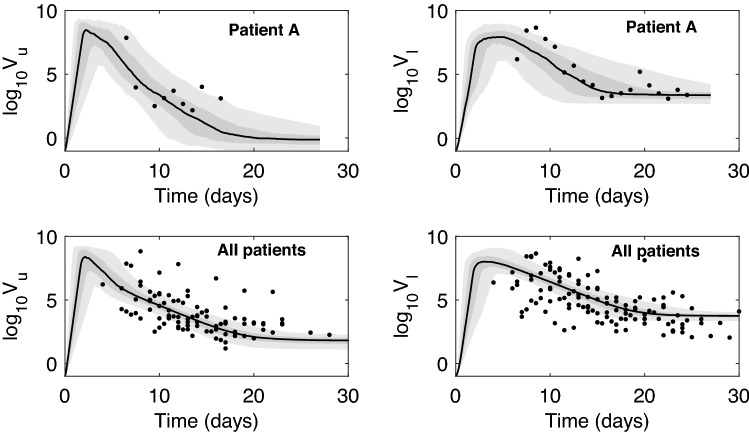
Virus dynamics obtained from fitting within-host model Eq. (7) to URT virus titer (left) and LRT virus titer (right) in patient A and in the entire population. The grey bars represent 50% and 95% posterior regions.

We generated prediction graphs of the within-host model Eq. (7) by sampling parameter realizations from posterior distributions. The model’s predictive posterior distribution for single patient URT-LRT viral data and population URT-LRT viral data are presented in Fig. 3. The resulting dynamics show viral expansion to peak values at days 2.1 in URT and 2.9 in LRT followed by decline in both tracts (see Fig. 3). The grey areas in the graph represent the 50% and 95% posterior regions. The fewer data points in patient A results in wider model prediction range (gray regions) compared to the population predictions, especially for the LRT viral load.

While the viral titers decay to low levels (below $10^2$ cp/ml) 3 weeks after infection in the URT, they stay elevated (to above $5.4\times 10^3$ cp/ml at week four) in the LRT. To model viral RNA persistence in the LRT we included a density dependent term for the loss of LRT virus, $c_l V_l/(V_l+K)$, and estimated parameter *K* where $V_l$ loss is half-maximal, together with the other viral specific terms.

We found similar mean infectivity rates in the URT for both the individual patient considered (patient A) and the entire population, $\beta _u=1.4\times 10^{-8}$ ml/(vir$\times$ day). By contrast, the mean infectivity rates in the LRT for patient A is 3.2-times higher than the LRT infectivity rate of the total population, $\beta _l=3.9\times 10^{-10}$ ml/(vir$\times$ day) versus $\beta _l=1.2\times 10^{-10}$ ml/(vir$\times$ day). The mean infected cells death rates are similar in URT and LRT, $\delta _u=4.9$, $\delta _u=4.6$ per day and $\delta _l=5.7$, $\delta _l=3$ per day for patient A and for the total population, respectively. The mean viral clearance rates are higher in LRT compared to URT, $c_l=11.5$, $c_l=9.2$ per day compared to $c_u=2.8$, $c_u=4.2$ per day, for patient A and for the total population, respectively. This may indicate increased immune responses occurring in LRT. The mean URT to LRT shedding rates are higher than the mean LRT to URT shedding rates, $k_u=0.24$, $k_u=0.63$ (swab/ml) per day compared to $k_l=7.9\times 10^{-4}$, $k_l=0.04$ (ml/swab) per day for patient A and for the total population, respectively. This one way shedding was observed by other studies that investigated the Wofle et al. data^3^. Lastly, the mean LRT viral load where viral clearance is half-maximal is $K=910$ RNA per ml for patient A and $K=1841$ RNA per ml for the total population.

### Practical identifiability

**Figure 4:**
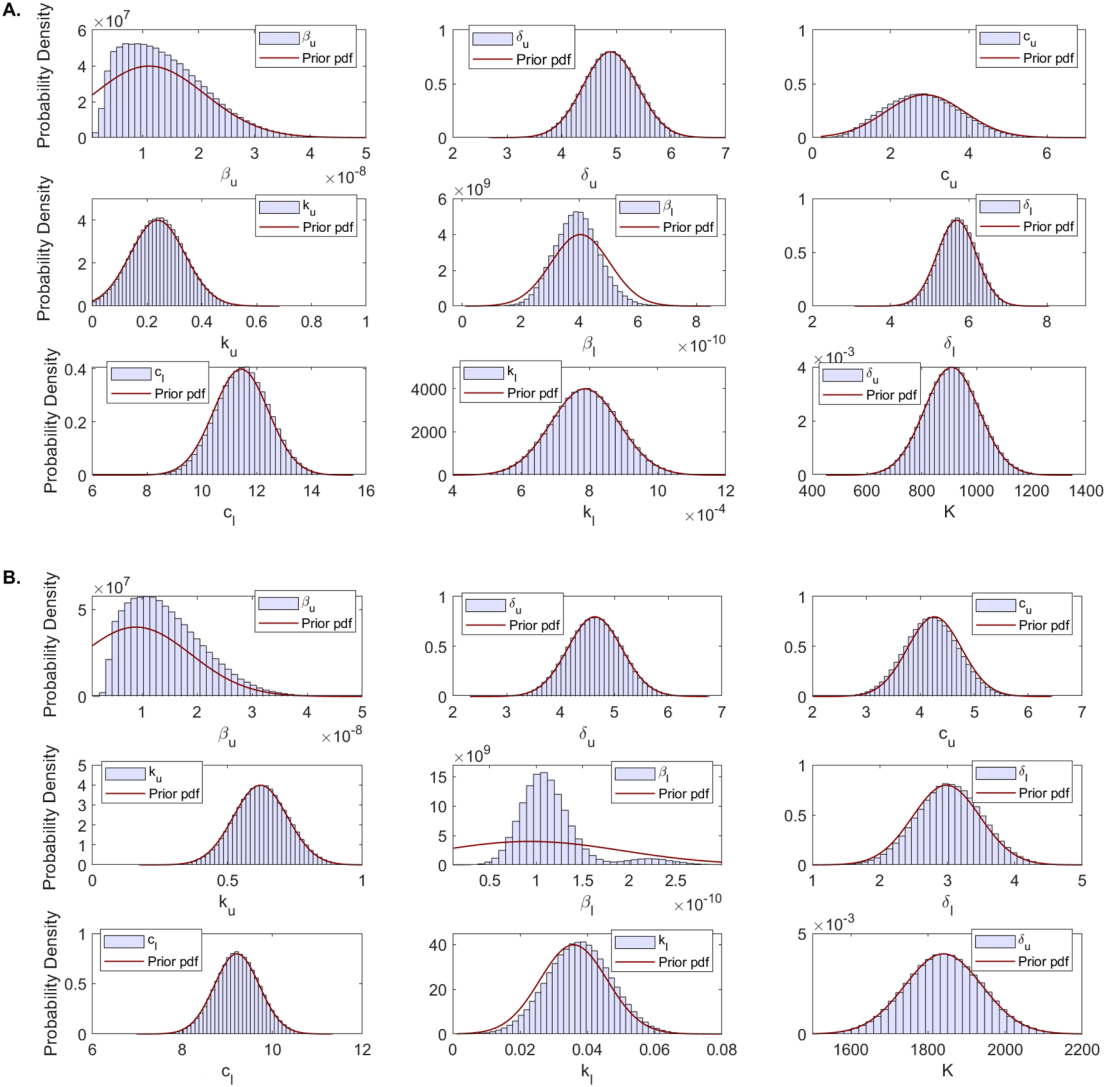
Histogram of estimated parameter distributions from fitting within-host model Eq. (7) to URT virus titer and LRT virus titer in: (**A**) patient A and (**B**) entire population. All parameters were considered normally distributed.

During MCMC data fitting, we used parameters limits predetermined to range around a single point estimation obtained using the ’fminsearch’ algorithm in Matlab (see Table 2). Parameter distributions for the nine parameter considered $p=\{\beta _u, \delta _u, c_u, k_u, \beta _l, \delta _l, c_l, K, k_l\}$ were obtained using an MCMC Bayesian approach that sampled the parameter space $N=10^6$ times. We apply DRAM MCMC algorithm and observe fast convergence of the chains (see Fig. 2). The resulting distributions, together with the prior probability density functions (pdf) are presented in Fig. 4. We observe good agreement between the prior pdf and the posterior distributions for all parameters with the exception of infectivity rates $\beta _u$ and $\beta _l$. Moreover, while all parameters follow normal distributions for patient A (Fig. 4A and Supplementary Fig. S1), the LRT infectivity rate $\beta _l$ follows a bimodal distribution in the fit to the total population data (Fig. 4B and Supplementary Fig. S2).

**Figure 5:**
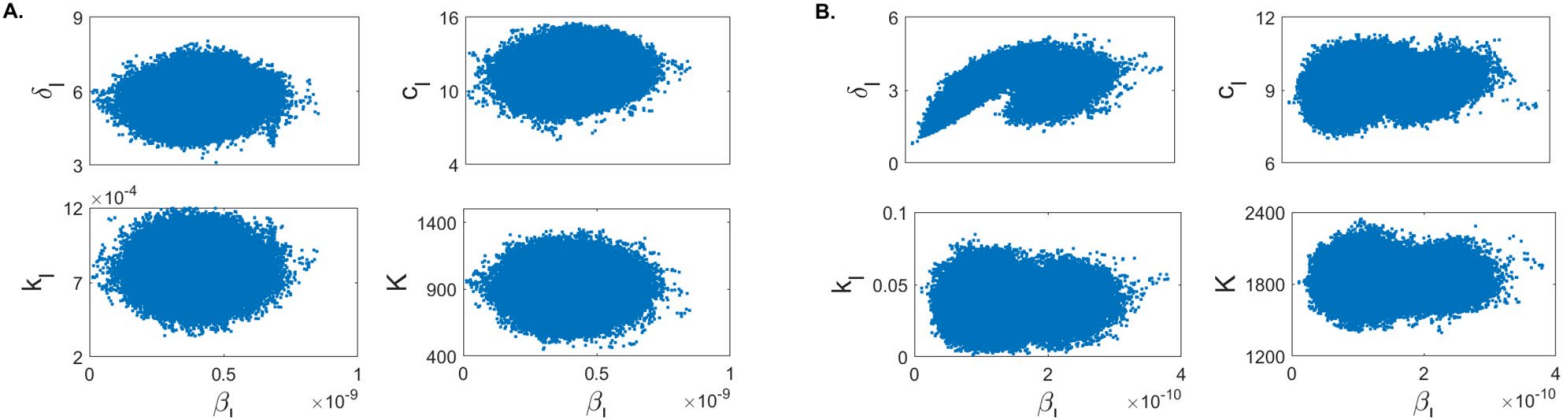
Scatter plots showing correlation among relevant parameters for (**A**) patient A and (**B**) total population.

Figure 5 shows the scatter plots of for paired $(\beta _l, k_l)$, $(\beta _l, K)$, $(\beta _l,\delta _l)$ and $(\beta _l, c_l)$ parameter distributions obtained when the within-host model Eq. (7) is fitted to patient A’s data (panel A) and population data (panel B) (see also Supplementary Fig. S2 for the scatter plots of all parameter distributions). In the scatter plots for the population data containing parameter $\beta _l$ we observe bimodal clustering. In joint density plots, bimodal clustering may suggest practical unidentifiability^40^. This implies that, despite the fact that we have shown that the within-host model Eq. (7) is structurally identifiable, it may in fact not be practically unidentifiable. It is well understood that a structurally identifiable model may be practically unidentifiable^28–30,41^. Many variables can lead to practical non-identifiability, such as considerable noise in the data, limited data points, or inadequate timing of data collection.

### Optimal experimental design

The possible lack of practical identifiability for the total population may be due to (1) restrictions on the parameter space and the types of distributions we are imposing on the parameters, or (2) the limited data points early in the infection.

**Figure 6:**
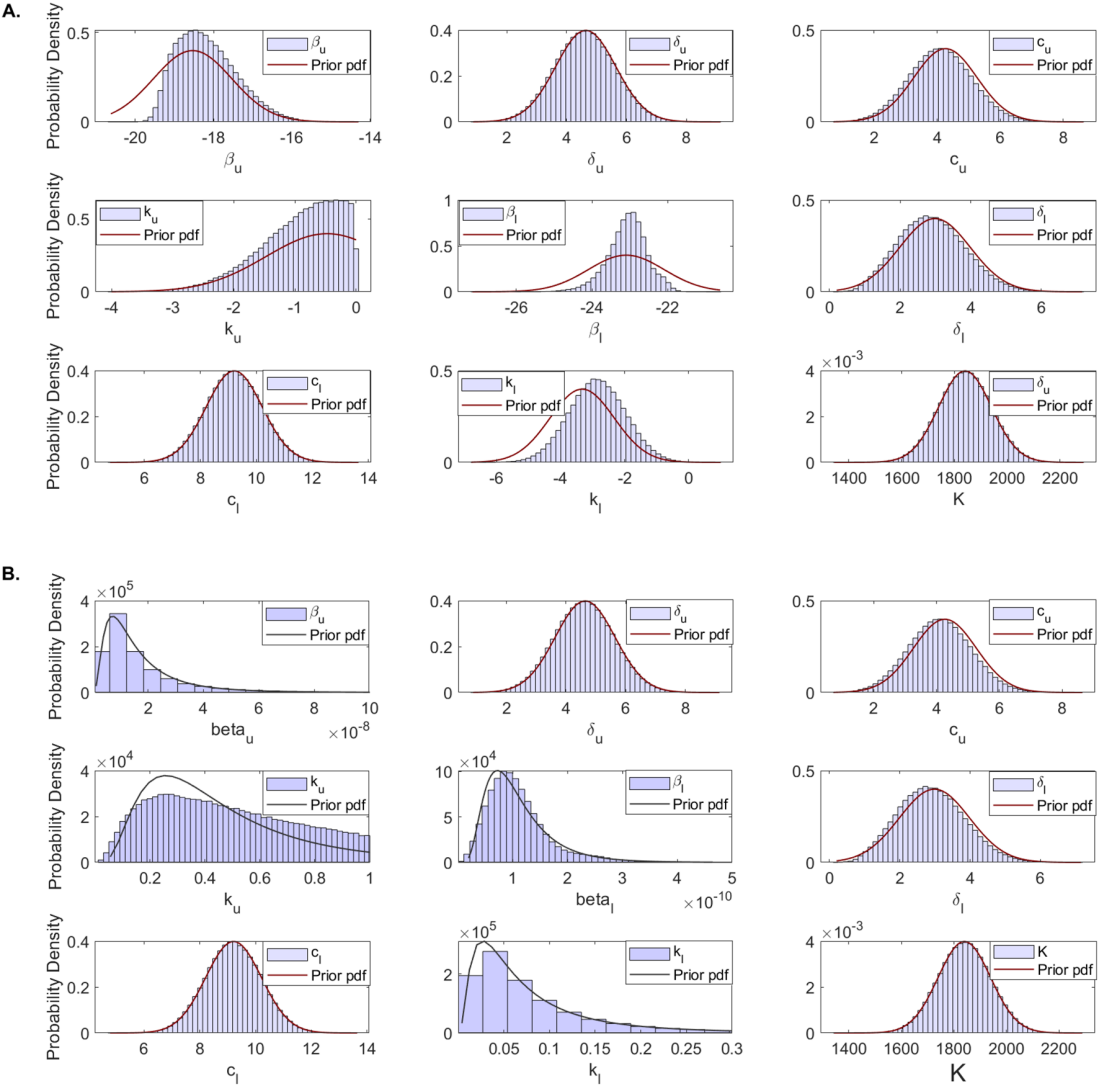
Histogram of estimated parameter distributions from fitting model Eq. (7) to URT virus titer and LRT virus titer in total populations. (**A**) Parameters $\ln \beta _u$, $\ln \beta _l$, $\ln k_u$, $\ln k_l$ were considered normally distributed. (**B**) Parameters $\beta _u$, $\beta _l$, $k_u$, $k_l$ were considered lognormally distributed. All other parameters were considered normally distributed.

To investigate the first hypothesis, we collected samples in the parameter space of

$$\begin{aligned} \{\ln \beta _u, \ln \beta _l, \ln k_u, \ln k_l\}, \end{aligned}$$

rather than $\{\beta _u, \beta _l, k_u, k_l\}$ and the assumed that either $\{\ln \beta _u, \ln \beta _l, \ln k_u, \ln k_l\}$ are normally distributed, or that $\{\beta _u, \beta _l, k_u, k_l\}$ are lognormally distributed. We set the limits of logarithmic parameter priors as in Table 3 while keeping the limits of the other parameters as before (see Table 2). We sampled the new parameter space $N=10^6$ times and reapplied the MCMC Bayesian approach. The resulting estimates for parameters $p=\{\beta _u, \delta _u, c_u, k_u, \beta _l, \delta _l, c_l, K, k_l\}$ no longer show bimodal results regardless on whether we assume that $\{\ln \beta _u, \ln \beta _l, \ln k_u, \ln k_l\}$ are normally distributed (see Fig. 6A) or that $\{\beta _u, \beta _l, k_u, k_l\}$ are lognormally distributed (see Fig. 6B).

**Table 3:** Adjusted parameters for the within-host model Eq. (7) are listed together with their lower and upper bounds for the priors which are normally distributed with mean equal to the fitted value and variance, $\sigma ^2$.

Parameter	Description	Prior $\pi (p)$	Population
		Min–Max	$\pi (p) \sim {\mathcal {N}} (\mu , \sigma )$
$\ln \beta _u$	Viral infectivity in URT (log scale)	$(-25, -14)$	${\mathcal {N}} (-18.5,1)$
$\ln \beta _l$	Viral infectivity in LRT (log scale)	$(-30, -15)$	${\mathcal {N}} (-23, 1)$
$\ln k_u$	Shedding into LRT (log scale)	$(-11, 0)$	${\mathcal {N}} (-0.5, 1)$
$\ln k_l$	Shedding into URT (log scale)	$(-7, 4)$	${\mathcal {N}} (-3.3, 1)$

To investigate the second hypothesis, we created synthetic data and used it to further examine how the timing of the data collection in the population correlates to the structure of the resulting parameter estimations. We assumed that the real data corresponds to the solution of model Eq. (7) with parameters in Tables 2 and 3 which are randomly perturbed according to Eq. (8) with errors $\epsilon _i$ and $\epsilon _j$ assumed to be uniformly distributed with mean 0 and standard deviation 0.5. We produced two data sets. The first data set, which assumes data is collected daily from day 0 to day 12 post infection, is

$$\begin{aligned} {\textbf {Experiment 1: }} \left( V_u^{data}(t_j), V_l^{data}(t_j)\right) \text { for } t_j=\{1, \ldots , 12\}. \end{aligned}$$

The second data set, which assumes data is collected from day 7 to day 27 post infection, is

$$\begin{aligned} {\textbf {Experiment 2: }} \left( V_u^{data}(t_j), V_l^{data}(t_j)\right) \text { for } t_j=\{7, \ldots , 27\}. \end{aligned}$$

**Figure 7:**
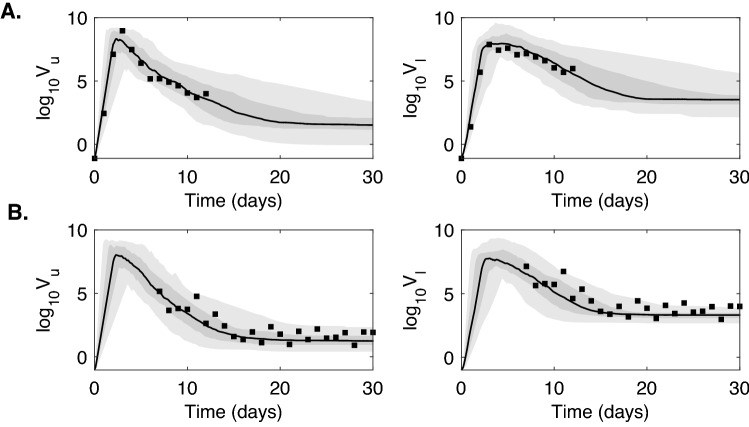
Virus dynamics obtained from fitting within-host model Eq. (7) to URT virus titer and LRT virus titer in (**A**) Experiment 1 and (**B**) Experiment 2. The grey bars represent 50% and 95% posterior regions.

Since the practical identifiability is a local property of the parameters, we used the priors for

$$\begin{aligned} p=\{\ln \beta _u, \ln \beta _l, \ln k_u, \ln k_l\} \end{aligned}$$

given in Table 3 and the priors for the rest of the parameters as in Table 2, to generate prediction graphs of the within-host model Eq. (7). The model’s predictive posterior distribution for all patients’ URT- LRT viral data for Experiments 1 and 2 are presented in Fig. 7 together with grey areas for the 50% and 95% posterior regions (see also Supplementary Fig. S4). As expected, we observe wider model prediction ranges (gray regions) in the second phase decay for experiment 1 and in the expansion and peak areas for experiment 2, where data is scarce (Fig. 7).

**Figure 8:**
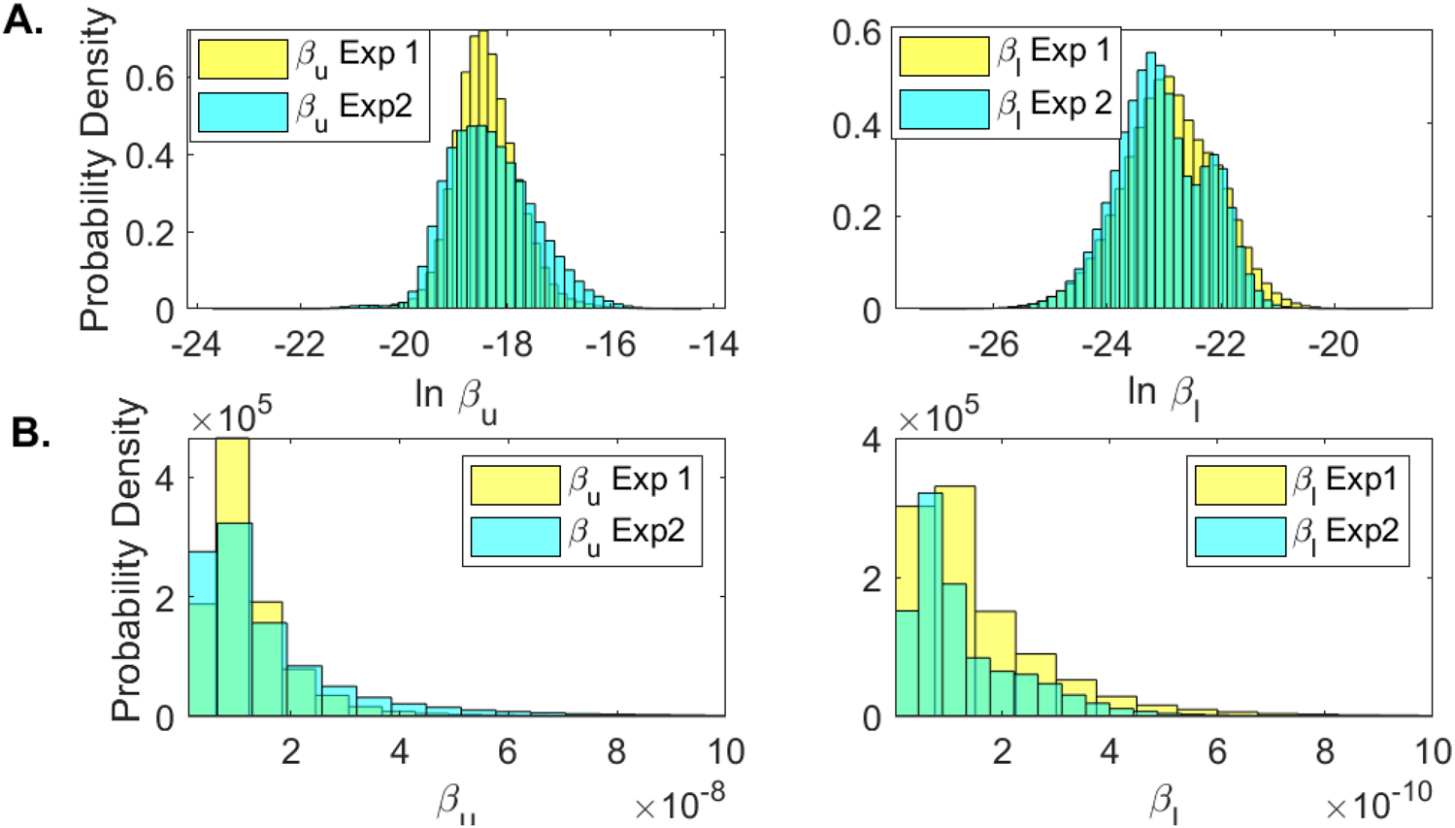
Histograms for $\beta _u$ and $\beta _l$ for Experiment 1 (yellow) and Experiment 2 (blue) when (**A**) $\ln \beta _u$ and $\ln \beta _l$ are assumed to be normally distributed; and (**B**) $\beta _u$ and $\beta _l$ are assumed to be lognormally distributed.

**Figure 9:**
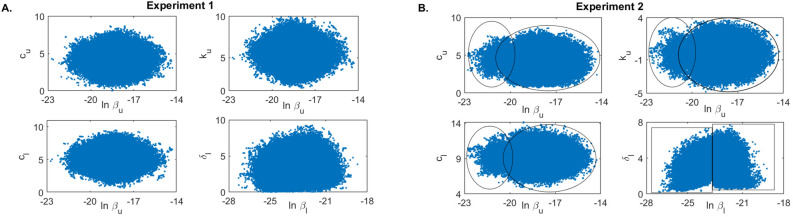
Scatter plots for (**A**) Experiment 1 and (**B**) Experiment 2. Parameters $\ln \beta _u$ and $\ln \beta _l$ are assumed to be normally distributed.

To determine whether practical identifiability is lost in each experiment we created parameter histograms for each parameters (see Fig. 8 and Supplementary Fig. S3). When data samples at the expansion stages of the infection are collected (as in Experiment 1), the LRT infectivity parameter $\beta _l$ follows a normal distribution (see Fig. 8A, left panel, blue bars). This results are validated by the corresponding dual parameter scatter plots (see Fig. 9A). In contrast, when the data at the expansion stages of the infection is sparse (as in Experiment 2), the LRT infectivity parameter $\beta _l$ follows a bimodal distribution (see Fig. 8A, right panel, blue bars). This results are observed in the corresponding scatter plots, where we see bimodal clustering involving not just parameter $\beta _l$, but involving parameter $\beta _u$ as well (see Fig. 9B). These results can be slightly improved when we consider that $\beta _u$ and $\beta _l$ follow lognormal distributions (see Fig. 8B, right panel, blue bars). This suggests that the practical unidentifiability that appeared in the population data might be fixed by collecting data at the early stages of infection.

## Discussion

In this study, we developed a within-host mathematical model of SARS-CoV-2 infection that connected the virus kinetics in the upper and lower respiratory tracts of infected individuals and used it to determine the tract specific viral parameters. We removed viral production rates, to ensure structural identifiability, and fitted the rescaled model Eq. (7) to published longitudinal throat swabs and sputum titers in a single individual and in the entire population from SARS-CoV-2 infection study^18^. We estimated nine unknown parameters using an MCMC Bayesian fitting approach^37^. To avoid over fitting, we determined best estimates in a single patient (for which we have 26 data points) and in the entire population (for which we have 201 data points). We found shorter virus life-spans in LRT compared to viral URT, 2–3 h compared to 5.7–8.5 h. Our LRT estimates are similar to the fixed (and non-tract specific) virus life-span of 2.4 h used by Ke et al.^3^ and the estimated (and tract specific) life-span of 1.2 h in Wang et al.^6^, but longer than the 10 h seen in influenza and used by Hernandez et al.^7^. The between tracts differences may suggest the presence of additional immune mediated viral clearance in the LRT. We found similar infected cells life-span between the two tracts, with a range of 4.2–8 h, shorter than in other studies^3,7^. Lastly, the mean URT basic reproductive number for the entire population, $R_0=\frac{\beta \hat{T_0}}{c \delta }$, equals 17.4, higher than in Ref.^3^. While we assumed two-way viral shedding between tracts, data fitting suggested higher virus shedding from upper and lower respiratory tracts than the other way around, consistent with other studies^3^.

Interestingly, we found that the estimated LRT infectivity rate parameter follows a bimodal distribution when the model was fitted to the entire population data. We attributed this behavior to practical non-identifiability. Practical non-identifiability is observed when the measured data is contaminated with noise. We have inherently accounted for noisy data by combining viral measurements from nine patients with different viral profiles. We investigated several ways for improving practical identifiability of this parameter and found that both estimating the logaritmic value $\ln \beta _l$ and assuming log-normally distributions for some parameters improves the accuracy of our estimates.

Most importantly, it has been reported that practical identifiability can be achieved by adding pertinent data measurements that can help improve the identity of unknown parameters^42,43^. Such a process, known as optimal experimental design, aims to obtain additional information about a system through the addition of new measurements. Since in system Eq. (7) the non-practically identifiable infectivity parameter $\beta _l$ is responsible for the LRT dynamics early in the infection, we investigated whether the addition of early data contains the maximal information needed for improving its estimate. We created two virtual data sets, one in which data is collected daily for the first 12 days and one in which data is collected daily for 20 days, starting at day 7. We found that the infectivity rate $\beta _l$ is bimodal and, hence, non-practically identifiable when data is missing during the first 7 days of infection. The absence of early data leads to an underestimation of overall LRT viral titer in the first 14 days following infection (see Supplementary Fig. S4). This may affect one’s ability for determining the best window for antiviral and immune modulation interventions^44^. Moreover, it will provide an underestimate for the period of maximum infectiousness^14^, which may affect recommendations for quarantine and isolation^1^. Hence, the existence of data measurements before and/or at symptoms onset is crucial for best parameter estimation and model prediction when considering noisy population data.

Our study has several limitations. First, we considered a density dependent clearance term for the URT virus that saturates at around 1–2 $\times 10^3$ RNA copies per ml, in order to explain the viral RNA persistence in the LRT at 30 days following infection reported in the Wolfle et al.^18^. While in public health setting a SARS-CoV-2 diagnostic is determined by PCR assays, long-term RNA levels are not a reliable measurement of infectiousness, with the measured RNA values indicating the presence of genomic fragments, immune-complexed or neutralised virus, rather than replication-competent virus^14,45,46^. Further work is needed to separate the presence of infectious versus non-infectious viral RNA in the lower respiratory tract. Secondly, we did not consider an eclipse phase in the virus infectiousness (usually assumed to be around 6 h^3,14^). This simplification may be the leading reason for larger infected cell death rate estimates in our study compared to other studies^3,7^. Thirdly, due to the novelty of the model, we have no information on parameter priors. Therefore, we fitted the within-host model to the patient A and population data, and used those estimates as means in the prior distributions. However, since the resulting means fall within ranges observed for other acute infections^23–26,47,48^, and since we consider large standard deviations around the prior means, we are confident that we are covering a large enough search space that does not exclude viable outcomes.

In conclusion, we have developed a within-host model of SARS-CoV-2 infection in the upper and lower respiratory tracts, used it to determine pertinent viral parameters, and suggested the optimal experimental designs that can help improve the model predictions. These techniques may inform interventions.

## Supplementary Information

Supplementary Information.

## Data Availability

The code generated during the current study will be made available on the corresponding author’s page upon acceptance.
