# Supplementary material for: Identifiability of parameters in mathematical models of SARS-CoV-2 infections in humans
Source: Sci Rep. 2022 Aug 27;12:14637. doi: 10.1038/s41598-022-18683-x (PMC9418662; doi:10.1038/s41598-022-18683-x)
Supplement: Supplementary file 1 — Supplementary Information. [file 41598_2022_18683_MOESM1_ESM.pdf]

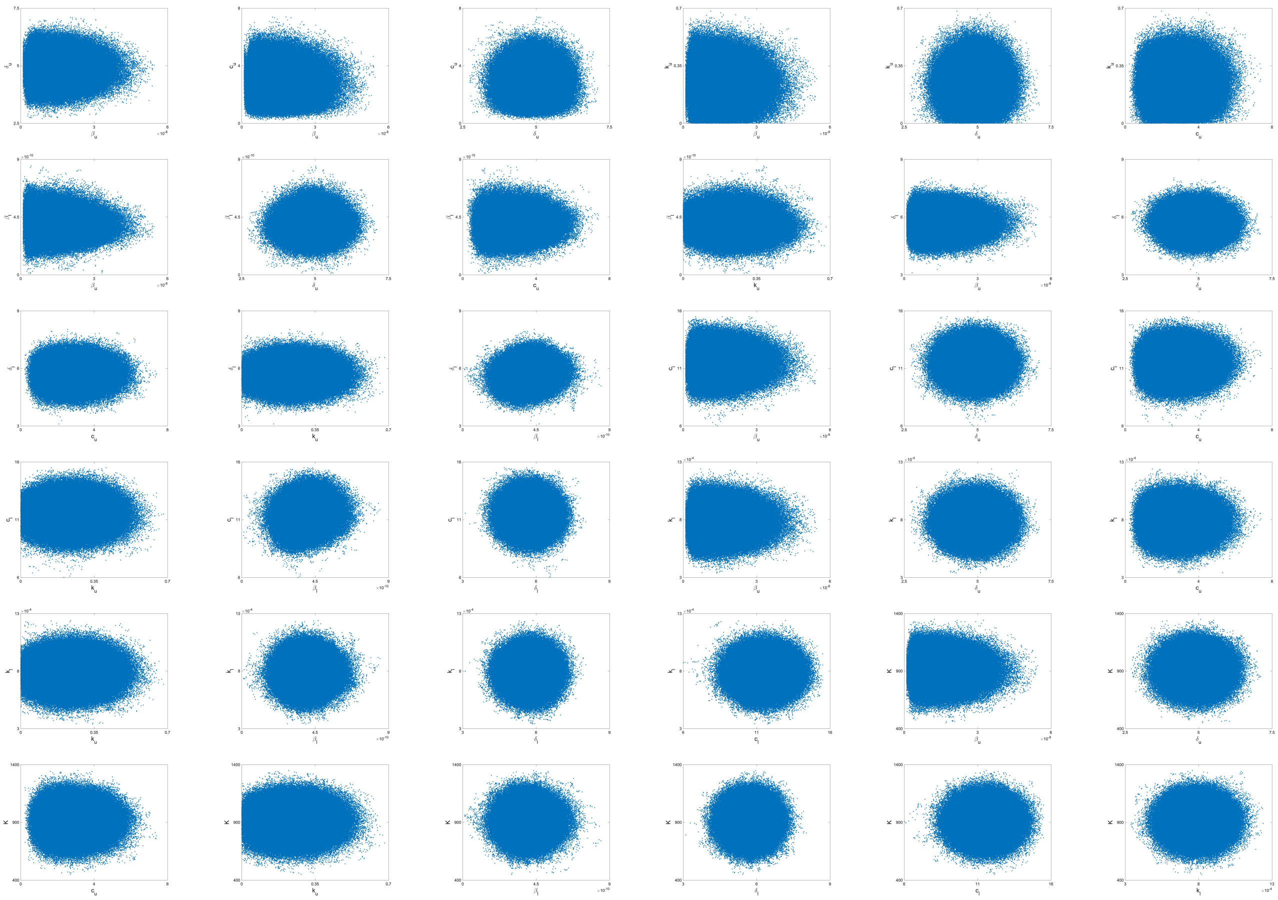

Figure S1. Two-parameter scatter plots for patient A. We sampled the parameter space $N = 10^6$ times.

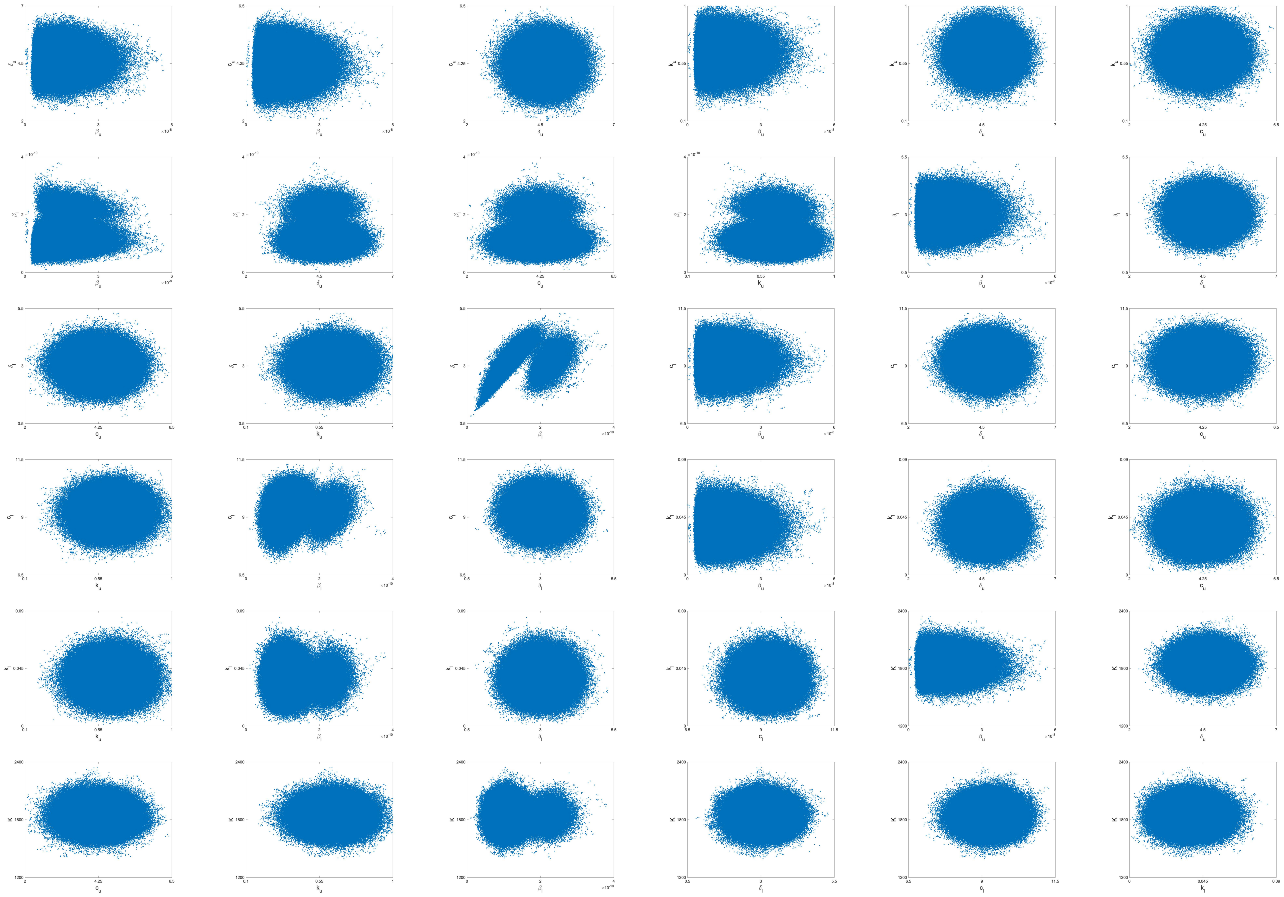

Figure S2. Two-parameter scatter plots for the total population. We sampled the parameter space $N = 10^6$ times.

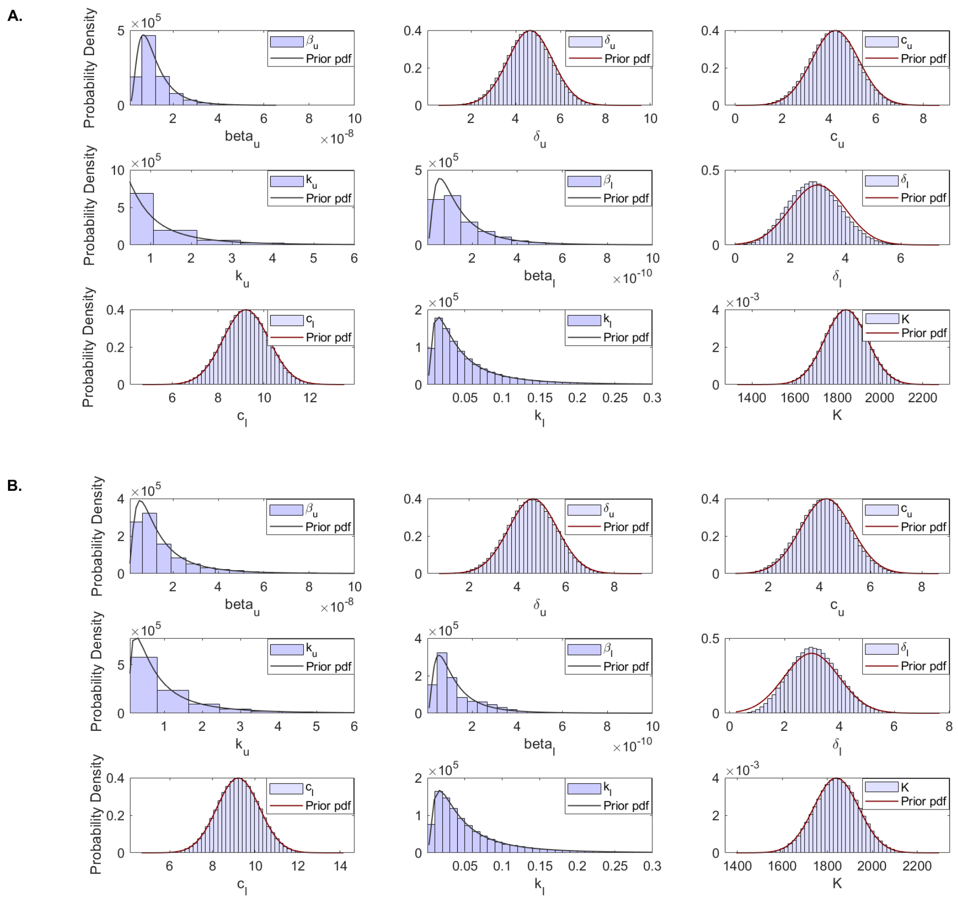

Figure S3. Histogram of estimated parameter distributions from fitting model Eq. (7) to virtual URT virus titer and LRT virus titer data in: (A.) Experiment 1 and (B.) Experiment 2. Parameters β_u, β_l, k_u, k_l were considered lognormal distributed. All other parameters were considered normally distributed.

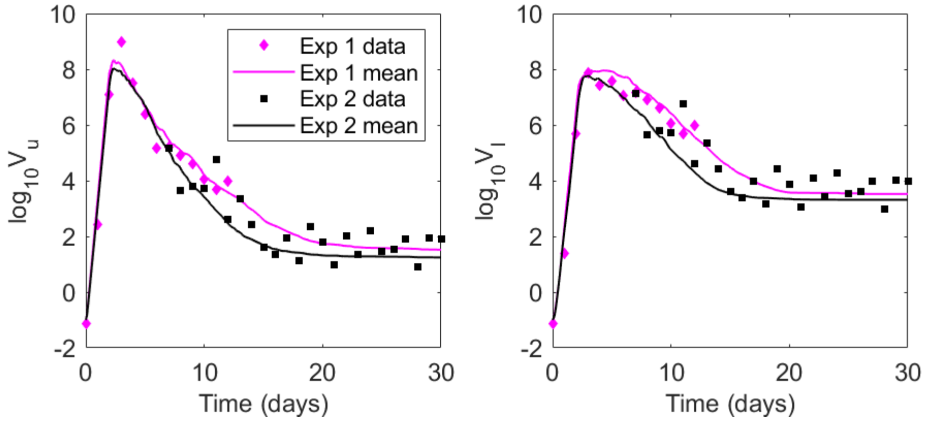

Figure S4. Virus dynamics obtained from fitting within-host model Eq. (7) to (left panel) URT virus titer and (right panel) LRT virus titer in Experiment 1 (magenta) and Experiment 2 (black).
